# Antimicrobial Stewardship in Urgent Care Settings: A Systematic Review of Prescribing, Clinical, and Microbiological Outcomes

**DOI:** 10.7759/cureus.114916

**Published:** 2026-08-21

**Authors:** Muhammad Amjad, John Morley, Rahma Fiaz

**Affiliations:** 1 Emergency Medicine, St. Luke's General Hospital, Kilkenny, IRL; 2 Emergency Medicine, Urgent Care Clinic Swords, Dublin, IRL; 3 Family Medicine, Dr Clinic, Kilkenny, IRL

**Keywords:** antibiotic prescribing, antimicrobial resistance, antimicrobial stewardship, clinical decision support, prescribing appropriateness, systematic review, urgent care, urgent care settings

## Abstract

Emergency and urgent care settings are frequently the first point of antimicrobial prescribing, making them a key setting for antimicrobial stewardship. Despite growing recommendations for implementing antimicrobial stewardship programs (ASPs) in urgent care settings, evidence of their effectiveness and impact remains dispersed. This systematic review evaluated the impact of ASPs on antimicrobial prescribing, clinical and microbiological outcomes, and implementation in urgent care settings. A systematic review was conducted in accordance with the Preferred Reporting Items for Systematic Reviews and Meta-Analyses (PRISMA) guidelines. MEDLINE (PubMed), EMBASE, Web of Science, and Scopus were searched from inception to identify eligible studies. Randomized and non-randomized studies, interrupted time series analyses, before-and-after studies, and cohort studies conducted in adult or pediatric urgent care settings were included. Two reviewers independently screened studies, extracted data, and assessed methodological quality. Owing to methodological and clinical heterogeneity, findings were synthesized narratively. Twelve studies published between January 2016 and March 2026 met the inclusion criteria. Most interventions combined clinician education, prescribing guidelines, clinical decision support, audits with feedback, pharmacist reviews, or multidisciplinary stewardship strategies. Eleven of the twelve completed studies reported reductions in antimicrobial prescribing or improved prescribing appropriateness following program implementation. Educational and guideline-based interventions demonstrated the largest reductions in broad-spectrum antibiotic use. Clinical outcomes, including mortality and return visits, remained largely unchanged. Evidence relating to microbiological and economic outcomes was limited, although *Clostridioides difficile* infection rates were generally stable or improved. ASPs improve the quality of antimicrobial prescribing in urgent care settings without adversely affecting patient outcomes. Greater standardization of stewardship interventions and outcome measures, together with robust multicenter studies evaluating microbiological and economic endpoints, is needed to strengthen the evidence supporting routine implementation in urgent care settings.

## Introduction and background

Antimicrobial resistance has increased steadily over recent decades and now represents a considerable burden on healthcare systems worldwide [1]. Infections caused by multidrug-resistant organisms are consistently associated with higher mortality, longer hospital stays, and markedly increased treatment costs [2]. It is now well recognized that inappropriate or excessive antimicrobial prescribing is a key factor behind the emergence and persistence of these resistant strains [3]. As a result, over the past two decades, antimicrobial stewardship programs (ASPs) have become central to public health strategy to limit the spread of multidrug-resistant bacteria and the infections they cause [4,5].

Where ASPs have been introduced across hospital settings, the available evidence indicates genuine reductions in antimicrobial consumption and improved patient outcomes [6]. However, their true effect on resistance rates remains uncertain [7,8]. Despite this uncertainty, professional and scientific bodies continue to strongly recommend their implementation [9].

To date, most published ASP experiences have focused on inpatient wards, particularly those managing critically unwell patients, with growing attention more recently turning towards primary care [6]. Urgent care settings, by contrast, remain a comparatively neglected setting within this body of literature, despite occupying a unique position in the stewardship landscape. Urgent care settings are typically where the first dose of an antibiotic is administered [10], and they are also responsible for a substantial share of antibiotics prescribed to patients discharged directly home or transferred to other care providers. Stewardship guidance widely acknowledges urgent care settings as a preferred setting for ASP implementation [11], yet whether such programs are actually being adopted and followed in day-to-day urgent care practice, rather than merely recommended in policy documents, remains far less clear.

Several barriers appear to distinguish urgent care settings from other hospital settings in terms of genuine adherence to stewardship principles [12,13]. The high patient turnover, the pressure to make empirical decisions without confirmed diagnostic results, and the limited presence of infectious disease specialists or pharmacists physically embedded within urgent care teams may all reduce the extent to which formal ASP recommendations are implemented at the point of prescribing [14]. Reports describing ASP structures in urgent care settings often focus on what programs intend to measure or achieve yet say comparatively little about whether front-line clinicians consistently follow the stewardship actions proposed, such as prospective audit and feedback, guideline adherence, or de-escalation once culture results become available. This distinction between an ASP existing on paper and an ASP being followed in clinical practice has received limited systematic attention.

Given the growing need to optimize antimicrobial use in urgent care settings, this systematic review aims to evaluate the effectiveness of ASPs implemented in these settings. Specifically, it examines the impact of stewardship interventions on antimicrobial prescribing and consumption, as well as their effects on clinical, microbiological, and economic outcomes. It also describes the range of stewardship strategies currently employed in urgent care settings and explores the available evidence supporting their effectiveness. By synthesizing the existing literature, this review provides a comprehensive overview of antimicrobial stewardship practices in urgent care and identifies important evidence gaps to guide future research and program development.

## Review

Search strategy and information sources

This systematic review was conducted in accordance with the Preferred Reporting Items for Systematic Reviews and Meta-Analyses (PRISMA) guidelines. A comprehensive literature search was conducted across four major electronic databases, namely MEDLINE (PubMed), EMBASE, Web of Science, and Scopus, from database inception to the search cut-off date. Search terms combined controlled vocabulary and free-text keywords based on three core concepts: antimicrobial stewardship, adherence or implementation, and the urgent care setting. These concepts were joined using Boolean operators, for example “antimicrobial stewardship” OR “antibiotic stewardship” AND “urgent care” OR “urgent care setting” AND “adherence” OR “compliance” OR “implementation”. Reference lists of relevant articles and previously published reviews on the topic were also hand-searched to identify additional eligible studies that the electronic search may not have captured.

Eligibility criteria

Studies were deemed eligible if they evaluated the implementation of or clinician adherence to an ASP in an urgent care setting, involving either adult or pediatric patients. Randomized controlled trials, non-randomized controlled trials, before-and-after studies, interrupted time series (ITS) analyses, and prospective or retrospective cohort studies were considered for inclusion. Studies were excluded if the principal intervention took place outside the urgent care setting, if no adherence or implementation outcome was reported, if the full text could not be obtained, or if the article was published in a language other than English or Spanish. Conference abstracts, editorials, commentaries, and narrative review articles were also excluded from the final synthesis.

Study selection process

Two reviewers independently screened all titles and abstracts against the predefined eligibility criteria using a standardized screening form. Any disagreement at this stage was resolved through discussion, and a third reviewer was consulted whenever consensus could not be reached. The full texts of potentially eligible articles were then retrieved and independently assessed in full by the same two reviewers. Where a full-text copy could not be located through institutional subscriptions or open access repositories, the corresponding author of the article was contacted by email and asked to provide the manuscript. Articles for which no response was received despite this correspondence were excluded from the review; this accounted for the exclusion of six articles.

Data extraction

A standardized data extraction sheet was used to record relevant study characteristics, including the first author, country of origin, year of publication, study design, sample size, patient population, components of the ASP, and the primary measure of adherence or implementation reported by each study. Data extraction was performed independently by two reviewers, and any discrepancies were resolved by mutual agreement.

Quality assessment

The methodological quality of each included study was appraised using a critical appraisal tool appropriate to its design, allowing studies at higher risk of bias to be identified and interpreted with appropriate caution during synthesis of the findings.

Study selection outcome

**Figure 1:**
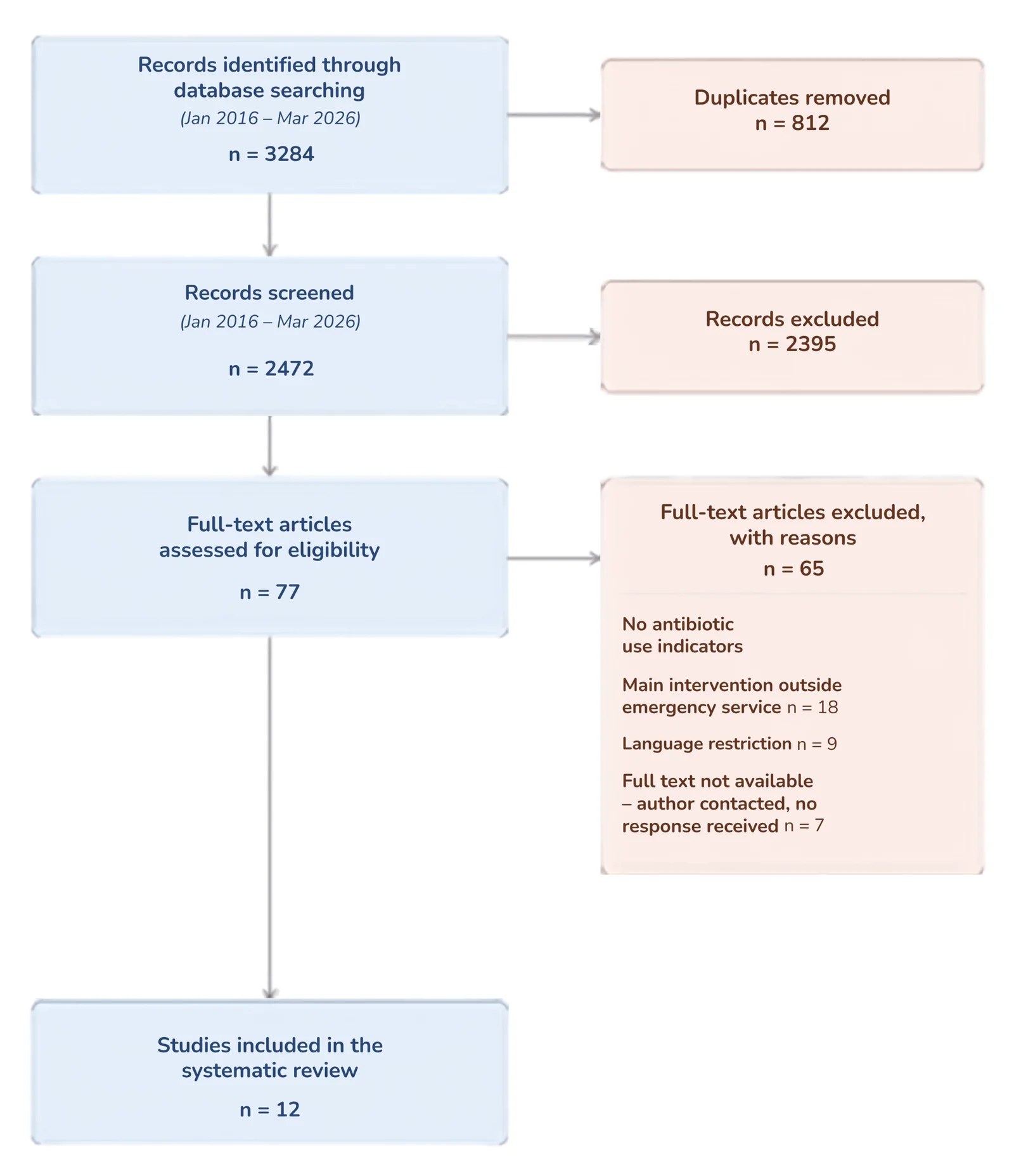
PRISMA flowchart illustrating the selection process of studies PRISMA: Preferred Reporting Items for Systematic Reviews and Meta-Analyses

The initial search strategy yielded more than three thousand records across the four databases. Following the removal of duplicate records and two stages of screening, 13 studies ultimately met the eligibility criteria and were included in the final qualitative synthesis of this systematic review. The complete study selection process is summarized in the PRISMA flow diagram below (Figure 1).

Risk of bias assessment

The risk of bias was independently assessed by two reviewers using the ROBINS E tool. This instrument evaluates seven domains, including confounding, exposure measurement, participant selection, post-exposure interventions, missing data, outcome measurement, and selective reporting. Each domain was judged as having a low, moderate, or high risk of bias. An overall risk-of-bias rating was then assigned to each study. Studies were considered to have a low overall risk when all domains were rated as low risk or when no more than two domains were rated as moderate risk. A moderate overall risk was assigned when all domains were rated as low risk except for one domain with a high risk of bias or up to three domains with a moderate risk. Studies with more than two domains rated as high risk or more than three domains rated as moderate risk were classified as having a high overall risk of bias.

Results

Overview of Included Studies

Twelve studies of ASPs in urgent care settings met the inclusion criteria for this review. They span 2016 to 2026 and are geographically weighted toward the United States, which contributed six studies; single studies came from Italy, Spain, Singapore, Ireland, France, and Canada. The studies employed a range of designs: retrospective and prospective quasi-experimental studies, ITS analyses, before-and-after cohorts, and quality-improvement studies built on front-line ownership (FLO) or plan-do-study-act (PDSA) cycles. One further study, a cluster-randomized trial protocol from Singapore, had not yet reported outcome data at the time of publication [15] and is described here but not analyzed further.

All studies were confined to urgent care settings. Populations ranged from small pediatric cohorts to adult, all-age, and mixed populations, and study sizes varied widely, from fewer than 400 patients to the 1.4 million urgent care visits reported by Guisado-Gil et al. [16]. Because the studies differ so much in design, in what was measured, and in how outcomes were defined, no attempt was made to pool results statistically. Findings are instead presented narratively, organized by outcome domain (Table 1).

**Table 1:** Descriptive characteristics of included studies ASP: antimicrobial stewardship program, CDI: *Clostridioides difficile* infection, CDS: clinical decision support, FLO: front-line ownership, ITS: interrupted time series, QE: quasi-experimental, QI: quality improvement, RCT: randomized controlled trial

Study	Design	Setting/population	Intervention	Follow-up	Risk of bias
Guisado-Gil et al., Spain 2024 [16]	QE-ITS	Urgent care observation unit (n = 238,876 transfers)	Education-based ASP (PRIOAM)	12 years (2011-22)	Moderate (no concurrent control)
Cunney et al., Ireland 2019 [17]	QI, FLO/PDSA	Pediatric urgent care	Weekly audit-and-feedback bundle	3 years (2014-17)	Moderate-high (small sample)
LaFave et al., USA 2019 [18]	Retrospective cohort	Urgent care-wide (~90,000 visits/y)	Urgent care sepsis order set (early broad-spectrum IV)	5 years	Moderate (protocol change)
Ford et al., USA 2023 [19]	QE-ITS	Urgent care + primary care clinics, all ages	CDS tool: azithromycin/fluoroquinolone alerts	~36 months	Moderate (COVID confound)
Dubrovskaya et al., USA 2023 [20]	Retrospective QE before-and-after	Urgent care discharge Rx, adults (n = 2,638)	Pharmacist review of discharge antibiotic Rx	30 days (8 weeks CDI)	Not assessed (abstract)
Barbieri et al., Italy 2020 [21]	Multi-center QE-ITS	3 pediatric urgent care (n = 3,039-3,980)	Clinical-pathway pocket-card algorithms	2014-19	Moderate (no control)
Kaufman et al., Canada 2017 [22]	QE, FLO-driven	Urgent care-wide (~167,000 visits)	Multi-pronged ASP (education, ID consult, DST)	2012-14	Moderate (confounded components)
Quintos-Alagheband et al., USA 2017 [23]	Prospective QI/PDSA	RSV bronchiolitis (n = 290)	Multifaceted ASP: guideline, audit/feedback	3.5 years	Moderate (concurrent QI initiatives)
Jorgensen et al., USA 2018 [24]	Retrospective before-and-after	Urgent care UTI discharges (n = 752)	Nitrofurantoin-first treatment algorithm	30 days	Moderate (unmatched cohorts)
Buehrle et al., USA 2021 [25]	Prospective ITS	VA urgent care discharge prescriptions	Education + monthly peer-comparison feedback	19 months	Moderate-high (no control, Hawthorne)
Yadav et al., USA 2019 [26]	Pragmatic cluster RCT	9 urgent-care sites (n = 44,820)	Education ± peer-comparison + public commitment	1 flu season	Moderate (no untreated control)
Ouldali et al., France 2017 [27]	Multicentric QE-ITS	7 pediatric urgent care (n = 242,534)	National ARTI antibiotic guidelines	5 years	Moderate (no untreated control)

Risk of Bias

Few studies applied a formal risk-of-bias tool. Where an assessment was reported, it typically landed at moderate or moderate-to-high risk, most often because the study lacked a concurrent control group, was conducted at a single center, drew on a small or non-random audit sample, or could not exclude a Hawthorne effect once staff knew they were being observed [17]. A handful of studies flagged more specific threats to internal validity: overlapping hospital-wide stewardship activity that could not be disentangled from the study intervention [17]; mid-study changes to the *Clostridioides difficile* infection (CDI) diagnostic protocol [18]; and, in two studies whose intervention periods coincided with the onset of the COVID-19 pandemic, the pandemic itself was entered into the model as a covariate in an attempt to reduce confounding (Table 1) [19,16].

Stewardship Interventions

Almost none of the interventions were single-component. Most combined two or more of the following: clinician education paired with prescribing guidelines; clinical decision support (CDS) tools that fired at the point of order entry; audit-and-feedback or peer-comparison reporting; prospective review by a pharmacist or infectious-disease specialist; and structured pathways or treatment algorithms. In practice, this took many forms: pharmacist review of urgent care discharge prescriptions [20]; azithromycin- and fluoroquinolone-specific CDS alerts [19]; pocket-card pathways for pediatric pharyngitis and acute otitis media [21]; an institution-wide education program known as PRIOAM [16]; FLO- or PDSA-driven audit and feedback [17,22,23]; a nitrofurantoin-first algorithm for urinary tract infection [24]; a sepsis order set that mandated early broad-spectrum therapy [18]; monthly peer-comparison feedback [25,26]; and dissemination of a national antibiotic guideline for acute respiratory tract infection [27].

Antimicrobial Use Outcomes

Of the 12 completed studies reporting an antimicrobial-use outcome (the Singapore protocol has none yet), eleven found less prescribing or consumption after the ASP was introduced. How much less depended heavily on the type of intervention and the antibiotic class in question. Education- and guideline-based approaches produced the largest effects: broad-spectrum prescribing for pharyngitis fell by 80%, and for acute otitis media by 29.5-55.2%, once clinical pathways were rolled out across three Italian pediatric urgent care settings [21]. A national guideline for acute respiratory tract infection cut overall prescribing by a cumulative 30.9% and broad-spectrum prescribing specifically by 62.7% [27]. An institution-wide education program sustained for more than a decade reduced total antimicrobial use by 45.6% relative to the expected trend, with individual agents falling by 34.5% (ceftriaxone) to 98.9% (metronidazole) [16].

Audit-and-feedback and decision-support approaches generally produced smaller reductions, though the changes were often still statistically significant. Weekly FLO-driven audit-and-feedback lifted documented-indication and guideline-compliance rates from 30% at baseline to 100%. At the same time, hospital-wide consumption dropped from 72.5 to 62.1 defined daily doses (DDD) per 100 bed-days [17]. A multi-pronged program combining infectious-disease consultation with urine culture optimization produced stepwise reductions in DDD per 1,000 urgent care visits for azithromycin, ceftriaxone, ciprofloxacin, and moxifloxacin [22]. Order-entry CDS alerts cut azithromycin prescribing by 24% in urgent care settings and by 47% in affiliated primary-care clinics almost immediately, with a slower, delayed decline in ciprofloxacin use in the clinics [19]. Peer-comparison feedback reduced inappropriate or antibiotic-nonresponsive prescribing in two further studies [25,26]. Pharmacist review of discharge prescriptions shifted the mix of antibiotic classes prescribed, with a physician acceptance rate of 92%, though the overall effect size was modest [20].

Two studies differed from this overall trend, and both did so deliberately. A nitrofurantoin-first algorithm for urinary tract infections increased nitrofurantoin prescribing from 16% to 43%. In comparison, cephalexin fell from 45% to 10%, not a reduction in prescribing overall, but a reallocation toward the guideline-preferred agent [24]. Likewise, a sepsis order set increased the proportion of urgent care visits receiving antibiotics from 33% to 38%; this was by design, since the order set aimed to initiate broad-spectrum treatment earlier for suspected sepsis rather than to curb use [18]. Taken together, these findings demonstrate that stewardship in urgent care settings does not always mean less antibiotic use; sometimes it means better-targeted use.

Clinical Outcomes

Secondary clinical outcomes were reported inconsistently, but where measured, they remained steady. The one clear treatment-failure signal ran in the intervention's favor: switching to nitrofurantoin as first-line UTI therapy was independently associated with fewer 30-day return visits (adjusted odds ratio, 0.547; 95% CI, 0.312-0.960) [24]. Fourteen-day mortality was numerically lower, though not significantly so, in the largest of the included studies (Table 2) [16].

**Table 2:** Antimicrobial-use outcomes and conclusions AMU: antimicrobial use, ARI: acute respiratory infection, ARTI: acute respiratory tract infection, BSI: bloodstream infection, CI: confidence interval, NS: not statistically significant, OR: odds ratio, RSV: respiratory syncytial virus, Rx: prescription/prescribing, AOM: acute otitis media, DDD: defined daily dose

Study	Primary antimicrobial-use outcome	Key clinical/microbiological outcome	Conclusion
Guisado-Gil et al. 2024 [16]	Total antimicrobial use −45.6% (95% CI −64.5 to −26.7)	BSI density +123.2%; mortality unchanged	Large use reduction; BSI signal warrants monitoring
Cunney et al. 2019 [17]	Guideline compliance 30% → 100% → 90%	Urgent care AMU 72.5 → 62.1	Sustained near-complete guideline compliance achieved
LaFave et al. 2019 [18]	Urgent care antibiotic-receiving visits ↑33% → 38% (by design)	Urgent care-wide CDI ↓11.6 → 6.2/10,000 pt-h	Earlier treatment intent met; CDI fell despite more use
Ford et al. 2023 [19]	Azithromycin urgent care −24%, clinics −47% (immediate change)	Not assessed	CDS alerts durably reduced targeted-agent prescribing
Dubrovskaya et al. 2023 [20]	2nd-gen cephalosporin 17% → 14% (p < 0.001); other classes NS	Urgent care LOS, 30-day revisit, CDI unchanged	Modest class-level shift; no downstream clinical effect
Barbieri et al. 2020 [21]	Broad-spectrum Rx ↓ 80% (pharyngitis), 29.5-55.2% (AOM)	Treatment failure unchanged	Pathway algorithms cut broad-spectrum use substantially
Kaufman et al. 2017 [22]	Stepwise ↓ DDD/1,000 visits (azithromycin, ceftriaxone, etc.)	Urgent care LOS	Reductions achieved without adverse operational impact
Quintos-Alagheband et al. 2017 [23]	Antibiotic exposure 56.2% → 30.9% (p < 0.001)	LOS unchanged; 30-day revisit NS	Multifaceted ASP cut RSV antibiotic exposure
Jorgensen et al. 2018 [24]	Nitrofurantoin 16% → 43% (p < 0.001)	30-day return 14.7% vs 19.9% (NS)	Shift to first-line agent without increased treatment failure
Buehrle et al. 2021 [25]	Overall Rx trend ↓ (NS); unnecessary Rx ↓ 55.6% → 38.7%	Not assessed	Reduced inappropriate prescribing; overall volume NS
Yadav et al. 2019 [26]	ARI Rx 6.2% → 2.4%; inappropriate Rx OR 0.67	Not assessed	Both intervention intensities reduced inappropriate Rx equally
Ouldali et al. 2017 [27]	ARTI Rx −30.9%; broad-spectrum −62.7%	14-day revisit unchanged	Sustained reduction with no rebound in treatment failure

Microbiological Outcomes

Five studies tracked CDI, and none reported an increase in CDI rates. Rates within eight weeks of a pharmacist-led review were unchanged [20], and no positive blood cultures were recorded during the intervention period for RSV bronchiolitis [23]. More striking is LaFave et al.'s finding [18]: nosocomial CDI fell from 11.6 to 6.2 per 10,000 patient-hours even as urgent care antibiotic prescribing rose, implying the CDI reduction was more attributable to the sepsis order set's earlier, better-targeted treatment than to any drop in overall exposure. The exception to an otherwise reassuring picture came from Guisado-Gil et al. [16], where a 45.6% fall in total antimicrobial use was accompanied by a significant rise in bloodstream infection incidence density (+123.2% relative effect), driven mostly by non-multidrug-resistant organisms, with resistance rates among the major Gram-negative and Gram-positive pathogens left largely unchanged. Diagnostic stewardship also had a measurable effect on testing behavior: urine-culture ordering fell significantly in the Canadian multi-pronged program (Table 2) [22].

Cost Outcomes

Only one study reported quantitative cost data. Cunney et al. reported that urgent care antimicrobial expenditure fell by roughly €105,000 in the year following implementation of their audit-and-feedback program, compared with the year before [17]. No other included study reported cost or expenditure data of any kind.

**Figure 2:**
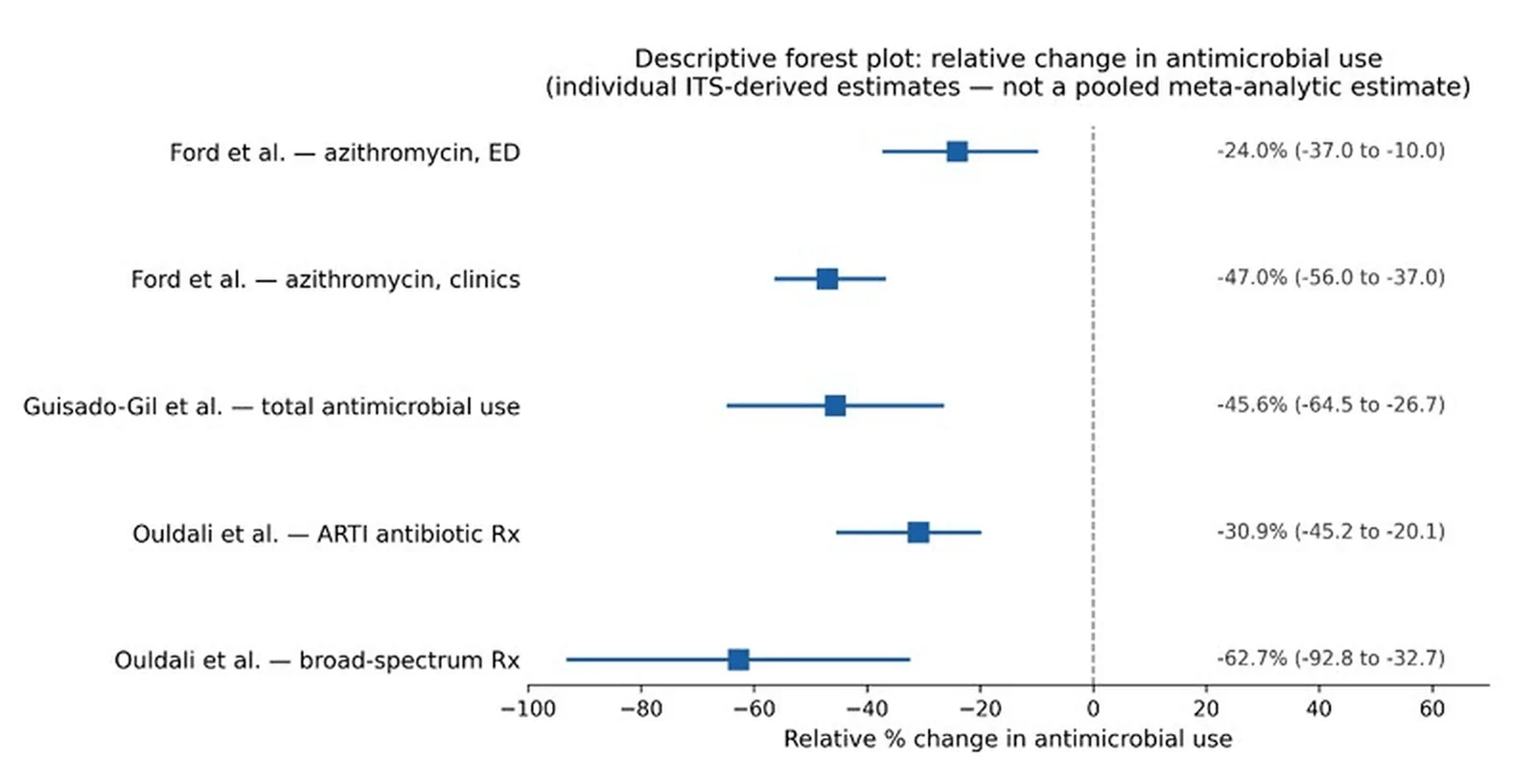
Relative change in antimicrobial use following ASPs across included studies Guisado-Gil et al. assessed long-term, education-based ASPs in an ED observation unit. Ouldali et al. evaluated the impact of national French guidelines on antibiotic prescribing for acute respiratory tract infections in a pediatric ED, reporting both the overall antibiotic prescription rate and the broad-spectrum antibiotic prescription rate as separate outcomes. ASPs: antimicrobial stewardship programs, ED: emergency department [16,19,27]

Figure 2 presents a descriptive forest plot of five ITS-derived effect estimates, plotted as relative % change, with the dashed line marking no effect. Differences in confidence interval widths are informative: Ouldali et al.'s broad-spectrum estimate carries a much wider CI than the others, reflecting a smaller subgroup analysis nested within their larger dataset [27].

Summary of Findings

Overall, ED-based stewardship interventions reduced antimicrobial prescribing or consumption in most of the studies reviewed here, with education and guideline-led programs, as well as class-specific decision support tools, delivering the largest effects. Two exceptions, both cases where higher antibiotic use was the intended outcome, reflecting earlier or more appropriate rather than reduced therapy, are a useful corrective to the assumption that stewardship success is always measured in less prescribing. None of the interventions studied worsened clinical outcomes such as mortality and return visits, and CDI rates remained steady or improved throughout. Evidence on microbiological and economic outcomes remains limited by comparison. The one study that examined resistance-relevant endpoints found a significant increase in the incidence of bloodstream infections alongside a decline in antimicrobial use. This finding argues for future ED stewardship research to incorporate microbiological and cost outcomes as standard, rather than treating antimicrobial-use figures as sufficient on their own (Figure 2).

A formal meta-analysis was not undertaken for this review. Of the 12 included studies conducted in urgent care settings, one [15] was a trial protocol with no results reported at the time of the review, precluding its inclusion in any quantitative synthesis. Among the 12, study designs were heterogeneous, spanning retrospective cohort studies, before-and-after quasi-experimental designs, ITS analyses, and quality-improvement reports using iterative PDSA methodology, with none conducted as randomized controlled trials. Populations also varied substantially in age (adult, pediatric, and mixed all-age urgent care settings) and clinical context (undifferentiated urgent care discharge prescribing, urinary tract infection, respiratory tract infection, RSV bronchiolitis, and sepsis pathways). Critically, antimicrobial-use outcomes were not reported on a common metric: studies variously expressed effects as proportions of prescriptions (%), DDD per 1,000 or 100 bed-days, odds ratios, or relative percentage change derived from segmented regression. This heterogeneity in design, population, and outcome operationalization meant that pooling effect estimates would not yield a statistically or clinically meaningful summary measure; therefore, a formal forest plot with a pooled estimate was not performed. A narrative synthesis structured by intervention type and outcome domain was adopted instead, consistent with Cochrane Effective Practice and Organization of Care guidance for reviews of heterogeneous quasi-experimental stewardship interventions. Where a subset of studies (Ford et al., Guisado-Gil et al., Ouldali et al.) [16,19,27] reported directly comparable relative-change effect estimates with 95% confidence intervals derived from ITS regression, these are displayed descriptively (Figure 2) to illustrate the range and precision of reported effects; this display does not represent a pooled or weighted meta-analytic estimate.

Discussion

The findings of this systematic review demonstrate that ASPs implemented in urgent care settings are consistently associated with improvements in antimicrobial prescribing. However, the magnitude of benefit varies according to the stewardship strategy, patient population, and clinical setting. Most included studies reported reductions in overall antibiotic prescribing or inappropriate antimicrobial use following implementation of stewardship interventions while maintaining favorable clinical outcomes [17,19,21-23]. These findings support current international recommendations that antimicrobial stewardship should extend beyond inpatient settings to include urgent care settings, where empirical antibiotic therapy is frequently initiated, and prescribing decisions substantially influence subsequent antimicrobial exposure and resistance patterns [28]. Recent guidance from the Infectious Diseases Society of America and the World Health Organization also recognizes urgent care settings as a priority setting for stewardship because they represent an important interface between hospital and community care [29]. Education-based interventions combined with locally developed prescribing guidelines demonstrated the greatest reductions in unnecessary antimicrobial use across the included studies [16,21,27]. The multicenter pediatric studies by Barbieri et al. and Ouldali et al. showed substantial declines in broad-spectrum antibiotic prescribing after the introduction of evidence-based clinical pathways and national prescribing guidance [21,27], without an increase in treatment failure or revisits. Similarly, the long-term education program reported by Guisado-Gil et al. achieved sustained reductions in total antimicrobial consumption over 12 years [16], suggesting that continuous educational reinforcement, supported by institutional commitment, may produce durable improvements in prescribing behavior [30,31]. These findings are consistent with previous studies reporting that educational strategies supported by standardized clinical guidance remain among the most effective antimicrobial stewardship interventions for improving prescribing quality in acute care settings [32-34].

Multifaceted stewardship programs incorporating clinical decision support [19], prospective audit [17,22,26], peer comparison [19], pharmacist review [20,22], and infectious diseases consultation [18,23,24] also demonstrated meaningful improvements in prescribing practice. Although reductions were generally smaller than those observed with structured educational programs, interventions involving electronic clinical decision support and audit-and-feedback consistently reduced inappropriate prescribing of targeted antibiotics such as azithromycin and fluoroquinolones [22,35,36]. These findings suggest that combining behavioral interventions with decision support tools may enhance adherence to antimicrobial guidelines while allowing clinicians to retain flexibility in complex urgent care presentations [37]. Similar conclusions have been reported in contemporary stewardship literature, where audit-and-feedback remains one of the most consistently effective behavioral interventions for improving antimicrobial prescribing [38,39].

Importantly, stewardship success in urgent care settings should not be interpreted solely as a reduction in antibiotic consumption [40]. Two included studies intentionally increased antibiotic prescribing in selected patient groups by implementing sepsis pathways and first-line urinary tract infection algorithms [18,24]. In these settings, stewardship promoted earlier administration of appropriate empirical therapy or greater use of guideline-recommended agents rather than restricting treatment [18,24]. This distinction reflects the fundamental objective of antimicrobial stewardship, namely, the optimization rather than the reduction of antimicrobial therapy. International stewardship frameworks increasingly emphasize appropriate antibiotic selection, timing, dosing, and duration rather than simple reductions in prescribing volume [41,42]. Clinical outcomes remained reassuring across the included studies. Urgent care return visits were generally unchanged following stewardship implementation, indicating that reductions in antimicrobial prescribing were not associated with adverse patient outcomes. Furthermore, the urinary tract infection pathway described by Jorgensen et al. was associated with fewer 30-day return visits [24], suggesting that improved adherence to guideline-recommended therapy may enhance treatment effectiveness while reducing unnecessary antimicrobial exposure [43]. These observations are consistent with evidence demonstrating that well-designed stewardship programs can safely optimize prescribing without compromising clinical care [44,45].

Evidence regarding microbiological outcomes was considerably more limited. Although several studies monitored CDI, no consistent increase was observed following stewardship implementation, and one study reported a reduction despite increased antibiotic prescribing associated with sepsis management [18]. Conversely, the increase in the incidence of bloodstream infections reported by Guisado Gil et al. highlights the complexity of interpreting ecological outcomes in urgent care settings, where multiple patient and organizational factors influence the epidemiology of infections [16]. Consequently, future urgent care stewardship programs should incorporate standardized microbiological surveillance alongside antimicrobial consumption measures to provide a more comprehensive assessment of program effectiveness. Similar recommendations have been proposed in recent international stewardship guidance, which advocates integrating antimicrobial utilization data with resistance surveillance and patient-centered outcomes [28].

Several important limitations are present in this systematic review. Most studies were conducted at single centers using quasi-experimental or before-and-after designs without concurrent control groups, increasing susceptibility to confounding and temporal bias. Considerable heterogeneity existed in stewardship interventions, outcome definitions, and antimicrobial consumption metrics, preventing quantitative synthesis and limiting direct comparison across studies. Furthermore, economic outcomes and resistance surveillance were infrequently reported despite their importance in evaluating stewardship effectiveness. Future research should prioritize multicenter, controlled studies that use standardized outcome measures and include microbiological, economic, and patient-centered endpoints to strengthen the evidence base for urgent care stewardship.

Overall, the available evidence indicates that ASPs implemented within urgent care settings improve prescribing quality without adversely affecting patient safety. Educational initiatives, clinical decision support, pharmacist involvement, and audits with feedback appear particularly effective when incorporated into multidisciplinary stewardship strategies. Establishing consensus regarding standard outcome measures, including antimicrobial consumption, microbiological surveillance, clinical outcomes, and healthcare costs, will improve comparability between studies and support wider implementation of effective stewardship programs across urgent care settings.

## Conclusions

ASPs implemented in urgent care settings consistently improve the quality and appropriateness of antimicrobial prescribing while maintaining favorable clinical outcomes. Educational interventions, clinical decision support, audit-and-feedback, and multidisciplinary stewardship approaches demonstrated the greatest benefits, although considerable variation in study design, stewardship strategies, and outcome measures limits direct comparison across studies. Evidence regarding microbiological and economic outcomes remains limited. Future research should adopt standardized stewardship indicators and incorporate clinical, microbiological, and cost outcomes to strengthen the evidence base and support effective implementation of ASPs in urgent care settings.
